# The Accuracy of Portable Monitoring in Diagnosing Significant Sleep Disordered Breathing in Hospitalized Patients

**DOI:** 10.1371/journal.pone.0168073

**Published:** 2016-12-19

**Authors:** Swamy Nagubadi, Rohit Mehta, Mamoun Abdoh, Mohammedumer Nagori, Stephen Littleton, Renaud Gueret, Aiman Tulaimat

**Affiliations:** Division of Pulmonary, Critical Care and Sleep Medicine, Cook County Health and Hospitals System, Chicago, Illinois, United States of America; Charite Universitatsmedizin Berlin, GERMANY

## Abstract

**Background:**

Polysomnograms are not always feasible when sleep disordered breathing (SDB) is suspected in hospitalized patients. Portable monitoring is a practical alternative; however, it has not been recommended in patients with comorbidities.

**Objective:**

We evaluated the accuracy of portable monitoring in hospitalized patients suspected of having SDB.

**Design:**

Prospective observational study.

**Setting:**

Large, public, urban, teaching hospital in the United States.

**Participants:**

Hospitalized patients suspected of having SDB.

**Methods:**

Patients underwent portable monitoring combined with actigraphy during the hospitalization and then polysomnography after discharge. We determined the accuracy of portable monitoring in predicting moderate to severe SDB and the agreement between the apnea hypopnea index measured by portable monitor (AHI_PM_) and by polysomnogram (AHI_PSG_).

**Results:**

Seventy-one symptomatic patients completed both tests. The median time between the two tests was 97 days (IQR 25–75: 24–109). Forty-five percent were hospitalized for cardiovascular disease. Mean age was 52±10 years, 41% were women, and the majority had symptoms of SDB. Based on AHI_PSG_, SDB was moderate in 9 patients and severe in 39. The area under the receiver operator characteristics curve for AHI_PM_ was 0.8, and increased to 0.86 in patients without central sleep apnea; it was 0.88 in the 31 patients with hypercapnia. For predicting moderate to severe SDB, an AHI_PM_ of 14 had a sensitivity of 90%, and an AHI_PM_ of 36 had a specificity of 87%. The mean±SD difference between AHI_PM_ and AHI_PSG_ was 2±29 event/hr.

**Conclusion:**

In hospitalized, symptomatic patients, portable monitoring is reasonably accurate in detecting moderate to severe SDB.

## Introduction

Sleep disordered breathing (SDB) is highly prevalent in patients with severe hypertension, heart diseases, stroke, and diabetes mellitus.[1–7] These conditions account for one sixth of hospitalizations in the US.[8] These hospitalizations may be opportunities to expedite the diagnosis and treatment for SDB and ultimately improve the health of these patients and reduce future healthcare costs.[9–14]

Once a hospitalized patient is suspected of having SDB, clinicians usually defer testing to the outpatient setting for many reasons. One reason is that a laboratory polysomnogram (PSG), the reference standard test, is not always feasible because hospitalized patients might have nursing needs that cannot be provided in the sleep laboratory or because the sleep laboratory might not have vacancy during the hospitalization. A second reason is that the conditions that lead to the hospitalization can change the severity of SDB.[15,16] Such a strategy, however, delays testing and might inadvertently deny it for patients who miss appointments.[10,17] Sharma and colleagues have advocated screening hospitalized patients based on symptoms and nocturnal oximetry to determine which patients should have an outpatient PSG.[18,19] This strategy still depends on patients showing up for their scheduled tests.[20]

An alternative approach to diagnose SDB during a hospitalization is performing portable monitoring (PM). PM has several advantages over PSG in hospitalized patients. It can be performed in the patient’s room without the need for transportation or interruption of care. Setting up a patient for PM requires minimal expertise because it does not include an encephalogram, oculogram, or electromyogram. PM also allows the performance of several studies simultaneously in contrast to PSG at the laboratory where one technologist can perform only two PSGs per night. Finally, the devices used for PM are much less expensive than those used for PSG. The main disadvantage of PM is the loss of data when a lead or a probe moves during a study, potentially making it uninterpretable.

In the inpatient setting, however, the use of PM raises two concerns. First is the general concern about the effect of acute illness on the severity of SDB. Second is the concern of underestimating the severity of SDB because sleep time, which is frequently reduced in hospitalized patients, is not usually measured during PM.[21–23] Accordingly, the American Academy of Sleep Medicine (AASM) in its most recent guideline does not recommend PM in patients with serious comorbidities.[23]

We undertook this prospective study to address the concerns about the accuracy of PM in hospitalized acutely ill patients in diagnosing SDB. We hypothesized that in hospitalized patients PM monitoring is accurate in detecting SDB that requires treatment. Hence, we determined the agreement between PM and PSG and the ability of PM to diagnose moderate and severe SDB.

## Methods

This study was conducted at the John H. Stroger, Jr., Hospital of Cook County, a large, urban public teaching hospital. It was approved by the Cook County Health and Hospitals System Institutional Review Board (IRB) and was registered at Clinical Trials.gov (NCT01424592). An informed IRB-approved written consent was obtained from subjects.

### Recruitment

Hospitalized patients were referred to the study during the hospitalization when their hospitalists suspected SDB. Participants were recruited from April 11, 2011 until Jan 11, 2012. Patients were excluded if they were previously diagnosed with SDB by PSG; if they were admitted to a surgical service; if they had septicemia, altered mental status, or an unstable psychiatric disorder; if they had facial deformities; or if they required contact, airborne, or droplet precautions.

### Study Protocol

Patients that consented to the study were interviewed. The Sleep Apnea Clinical Score (SACS) and the Epworth Sleepiness Scale (ESS) were calculated.[24,25] The presence of diabetes mellitus, hypertension, cardiovascular diseases, chronic obstructive pulmonary disease, and reason for admission were ascertained by review of the medical record. Height, weight, and neck circumference were measured. Each patient had a PM study during the hospitalization and was scheduled for an outpatient PSG.

### Portable Monitoring

The study protocol did not specify criteria for when to perform the PM study. The investigators and the referring hospitalists determined the optimal time to perform the PM study, which occurred when the patient was medically stable based on the clinical judgement of the referring hospitalist and investigator (SN) and preferably the day before discharge.

An Alice PDx device (Philips Respironics, Murrysville, PA) was used to collect simultaneously airflow (nasal pressure cannula and oral thermistor), effort (chest and abdominal movement with two respiratory plethysmography belts), pulse oximetry with a finger probe, and sleep (wrist actigraphy). Each patient’s nurse and medical resident were asked to minimize the use of oxygen, given information about how to troubleshoot the device, and instructed to call the investigators for any questions. An investigator set up the PM equipment on each patient on the night of the study in the patient’s hospital room.

One blinded, board-certified sleep specialist (SN) scored all PM studies. Each PM study was reviewed to confirm that it included at least 3 hours of recording time. The investigator used the actigraphy to select the beginning and end of the PM study. The selected segment was reviewed in 30-second epochs. The epochs were staged either as wake or sleep using analysis of actigraphy combined with motion artifact from the other leads. Apnea was defined as a cessation of airflow during sleep for more than 10 seconds. An apnea was deemed obstructive if there were respiratory efforts throughout it and central if there was no respiratory effort. Hypopnea was scored if there was at least a 10 second discernible reduction in flow associated with a 4% desaturation. If there was loss in the flow signals, the effort channel was used for scoring the events as recommended in the technical specifications for portable monitoring in the AASM scoring manual.[26]

### PSG

Before discharge, all patients were scheduled for outpatient PSGs (Somnostar z4, Carefusion, Vernon Hills, Illinois) at the hospital’s AASM accredited sleep laboratory. During the PSG the following were recorded: 6 EEG channels, 2 electroocculogram channels, 3 EMG channels, 2 airflow channels (oronasal thermistor and nasal pressure transducer), electrocardiogram, thoracic motion, abdominal motion, snoring, and finger pulse oximetry.

CPAP was initiated during the PSG (split-night PSG) after two hours of sleep if the apnea hypopnea index (AHI) was ≥ 15 events per hour based on the visual impression of the polysomnography technologist. PSGs were scored according to the “acceptable” AASM hypopnea criteria that requires a 4% desaturation to score a hypopnea.[26] The PSGs were reviewed by board certified physicians blinded to the results of the PM studies.

### Data Analysis

Sample size calculation was based on the area under the ROC curve. We assumed that the acceptable area would be 0.85, the null hypothesis for the area is 0.70, an alpha 0.05, and a beta of 0.1, the ratio of no and mild SDB to moderate and severe SDB of 0.5. The required sample would be 100 patients. We assumed that one third of the patients will miss the PSG appointment; therefore we aimed to recruit about 150 patients.

Variables are reported as mean ± standard deviation (SD) or interquartile range (IQR 25–75) as indicated. Respiratory indexes were calculated by dividing the number of events by the total sleep time measured by EEG in PSG and by actigraphy in PM. For example, the apnea hypopnea index with PSG (AHI_PSG_) and with PM (AHI_PM_) was calculated by dividing the number of apneas plus hypopneas by sleep time. Two cutoffs were used to identify patients with significant SDB: moderate or severe SDB, AHI_PSG_ ≥ 15; and severe SDB, AHI_PSG_ ≥ 30. Significant central sleep apnea (CSA) on PM was defined as central apnea index (CAI_PM_) ≥ 5. We calculated AHI_PM_—CAI_PM_ for each PM study as a measure of non-central SDB.

Our objective was to determine the ability of PM to identify patients with significant SDB. The area under the receiver operator characteristic (AU-ROC) curve was calculated to determine the accuracy of PM. We identified the optimal threshold for AHI_PM_ to diagnose SDB by performing a bias-corrected bootstrapping estimation of the Youden Index ([sensitivity+specificity]-1).[27] We calculated the sensitivity and specificity, positive and negative likelihood ratios of the various AHI_PM_ thresholds. The relation between AHP_PSG_ and the respiratory indexes by PM was determined with linear regression.

The correlation between AHI_PM_ and AHI_PSG_ was explored by calculating the Spearman and the intraclass correlation coefficients. Modified Bland-Altman plots were constructed to assess the agreement between AHI_PM_ and AHI_PSG_. The mean of the difference between AHI_PM_ and AHI_PSG_ (bias) and the limits of agreement (1.96SD of the mean of the differences) were calculated.

Statistical analysis was performed using Medcalc (version 12.7 MedCalc Software, Ostend, Belgium).

## Results

### Patient Enrollment and Follow-up

One hundred and fifty-seven consecutive patients suspected of having SDB were referred to the study. Five patients were excluded: 1 had sepsis and 4 patients had PSG in the past. One hundred and fifty-two patients were tested during the hospitalization with PM. Only 72 patients had PSGs after discharge. The remaining patients missed their PSG appointments despite attempts to reschedule them. The median time between the two studies was 97 days (IQR 25–75: 24–109). All PSG recordings were adequate. Four PM recordings were discarded: 2 for short recording and 2 for device failure. Only one of these four underwent PSG. Hence, 71 patients were included in the final analysis (Table 1). Seven PM studies were performed on oxygen by nasal cannula. Only two patients had less than four hours of acceptable recording. One of these studies had less than three hours of recording with pulse oximetry, flow, and effort; it was scored mostly by relying on oximetry and effort.

**Table 1:** Patient Baseline Characteristics.

Age, years†	52 ± 10
Women, n (%)	29 (41)
BMI, kg/m^2^	39 (35–46)
Neck circumference, cm	44 (42–48)
Epworth Sleepiness Scale score	14 (10–18)
Nights per week with loud snoring	7 (6–7)
Nights per week with gasping	6 (4–7)
SACS†	30 (22–66)
Admission for cardiovascular Diagnosis, %	45%
Comorbidities:	
Hypertension, %	94
Diabetes mellitus, %	38
Obstructive pulmonary disease, %	18
Ejection fraction (n = 48), % †	53 ± 13
Laboratory:	
PCO_2_, mmHg (n = 46)	51 (44–57)
Bicarbonate, mEq/L	28 (26–32)
HGB_A1C_, % (n = 40)	7.2 (6.5–8.1)
Highest troponin-I, ng/mL (n = 58)	0.017 (0.012–0.033)
Delay between PM and full PSG, d	106 (75–110)
Delay between PM and split PSG, d	73 (21–109)

^†^: mean ±SD.

All other variables were in median (IQR 25—IQ75) or percentage as indicated.

### Comorbidity

Most patients had hypertension. Ten patients had systolic heart failure with an ejection fraction ≤ 40%. Arterial blood gases were measured in 46 patients based on the hospitalist’s discretion and at various times in the hospitalization. Thirty-four patients had hypercapnia (PCO_2_ > 45 mmHg). Almost 20% had a diagnosis of obstructive lung disease. Diabetes mellitus was also common (Table 1). Patients were discharged with the following diagnoses: 16 with congestive heart failure, 14 with non-cardiac chest pain, 8 with coronary artery disease, 6 with edema, 5 with uncontrolled hypertension, 5 with hypercapnic respiratory failure, 4 with obstructive pulmonary disease, 3 with pulmonary hypertension, 2 atrial arrhythmia, 8 with other diagnoses.

### Sleep Disordered Breathing

Based on actigraphy, the sleep time during PM studies was more than 300 minutes in most patients and the sleep efficiency was similar to the sleep efficiency with PSG (Table 2). Fifty-three patients had an AHI_PM_≥15 and thirty-nine had an AHI_PM_≥30.

**Table 2:** Sleep variables from PM and PSG based on the type of outpatient PSG.

Variable	Patient with full night PSG (n = 27)		Patients with split-night PSG (n = 44)	
	PM	PSG	PM	Split PSG
Sleep time, minutes	413 (308–476)	320 (269–363)	472 (392–501)	117 (89–134)
Sleep efficiency, %	84 (70–92)	80 (70–85)	90 (84–94)	80 (58–89)
AHI	19 (10–35)	9 (6–14)	51 (25–85)	64 (35–93)
OAI	1 (0–3)	1 (0–2)	6 (2–15)	16 (3–39)
CAI	0 (0–2)	0 (0–0)	1 (0–2)	0 (0–1)
Lowest O_2_ saturation, %	78 (69–85)	78 (68–83)	72 (61–80)	74 (66–81)
Percent of sleep time with saturation<90%	7 (1–50)	16 (5–73)	22 (6–46)	57 (31–95)

All variables are medians (IQR 25–75).

OAI = obstructive apnea index.

AHI: apnea hypopnea index.

CAI: central apnea index.

Forty-four patients had split-night PSG and twenty-seven patients had full night PSG (Table 2). Sixty-five patients were found to have SDB (AHI_PSG_≥5): mild (AHI_PSG_ 5–14) in 17, moderate (AHI_PSG_ 15–29) in 9, and severe (AHI_PSG_ ≥30) in 39.

**Fig 1:**
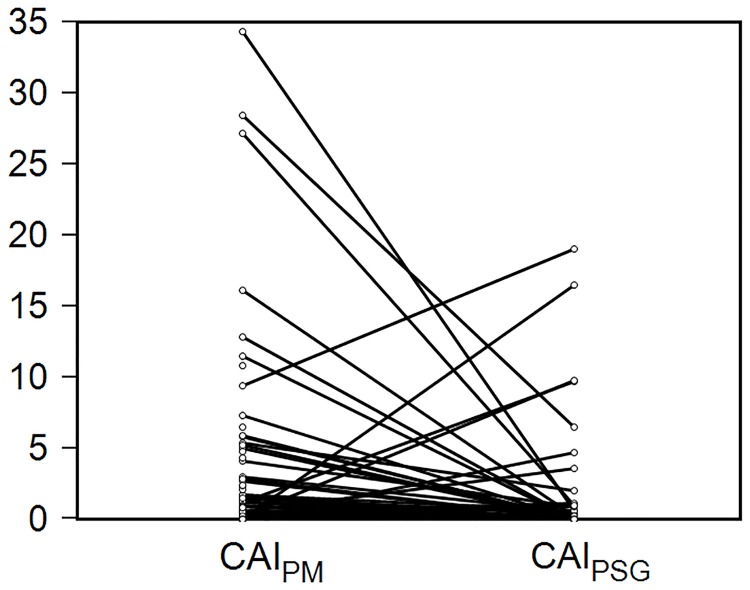
The central apnea indices on portable monitoring and polysomnography. The central apnea index with the portable monitor (CAI_PM_) during the hospitalization was generally higher than on outpatient polysomnography (CAI_PSG_).

The CAI_PM_ was higher than CAI_PSG_ (3±7 versus 1±3, P = 0.04) (Fig 1). CAI_PM_ was ≥5 in 13 patients. In the 48 patients with known left ventricular ejection fraction, the difference between CAI_PM_ and CAI_PSG_ was higher (5±10) in patients with low ejection fraction (n = 11) than in patients with normal ejection fraction (1±5), p = 0.03.

#### Predicting patients with significant SDB

**Fig 2:**
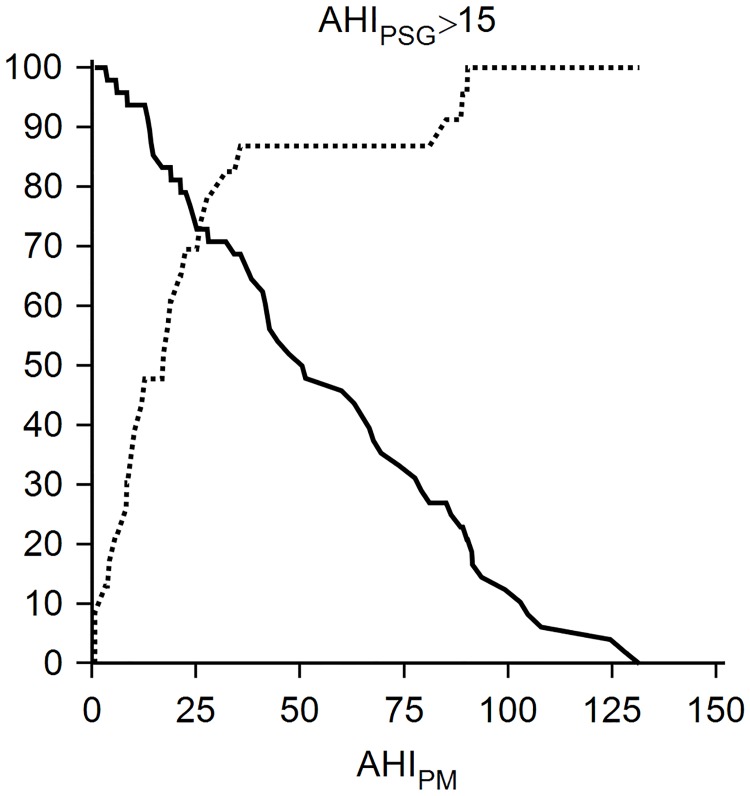
Plot versus criterion graph. This graph plots the sensitivity and specificity with 95% confidence intervals for different cutoff values of AHI_PM_; the criterion was AHI_PSG_ ≥ 15.

AHI_PM_ and (AHI_PM_—CAI_PM_) had similar AU-ROC curve in predicting AHI_PSG_ ≥ 15. The optimal threshold for both variables was 36 (Table 3). The AHI_PM_ threshold with 90% sensitivity was 14, which had a specificity of 48% (Fig 2). The AU-ROC curve was similar between patients with ESS higher and lower than the median ESS and was also similar between patients with neck circumference higher and lower than the median neck circumference. Likewise, AHI_PM_ and (AHI_PM_—CAI_PM_) had similar AU-ROC curve for predicting AHI_PSG_ ≥ 30. The optimal AHI_PM_ thresholds were 22 and 19, respectively (Table 3).

**Table 3:** Accuracy of PM in predicting AHI_PSG_ > 15 and > 30.

	Predicting AHI_PSG_ ≥ 15		Predicting AHI_PSG_ ≥ 30	
	AHI_PM_	AHI_PM_—CAI_PM_	AHI_PM_	AHI_PM_—CAI_PM_
AU-ROC	0.80	0.81	0.82	0.84
Threshold	36	36	22	19
Sensitivity	69%	69%	87%	92%
Specificity	87%	87%	66%	66%
PPV	92%	92%	76%	77%
NPV	57%	57%	81%	88%
+LR	5.3	5.3	2.5	2.7
-LR	0.36	0.36	0.2	0.12

PPV: positive predictive value. NPV: negative predictive value. LR: likelihood ratio.

#### The accuracy of PM in patients with and patients without hypercapnia

In our sample there were 31 patients with PaCO_2_> 45mmHg and 23 of them had an AHI_PSG_ > 15. The AU-ROC for AHI_PM_ predicting AHI_PSG_≥ 15 was 0.88 with an optimal threshold of 19 (sensitivity of 91% and specificity of 75%).

There were 40 patients with PaCO2≤ 45 mmHg or without measurement of arterial blood gases. The AU-ROC for AHI_PM_ predicting AHI_PSG_≥ 15 (25 patients) was 0.73 with an optimal threshold of 32 (sensitivity of 64% and specificity of 87%). The AU-ROC for AHI_PM_ predicting AHI_PSG_≥ 30 (20 patients) was 0.82 with an optimal threshold of 32 (sensitivity of 75% and specificity of 85%).

#### Comparison of AHI_PM_ to clinical parameters in predicting significant SDB and the role of central apneas

**Fig 3:**
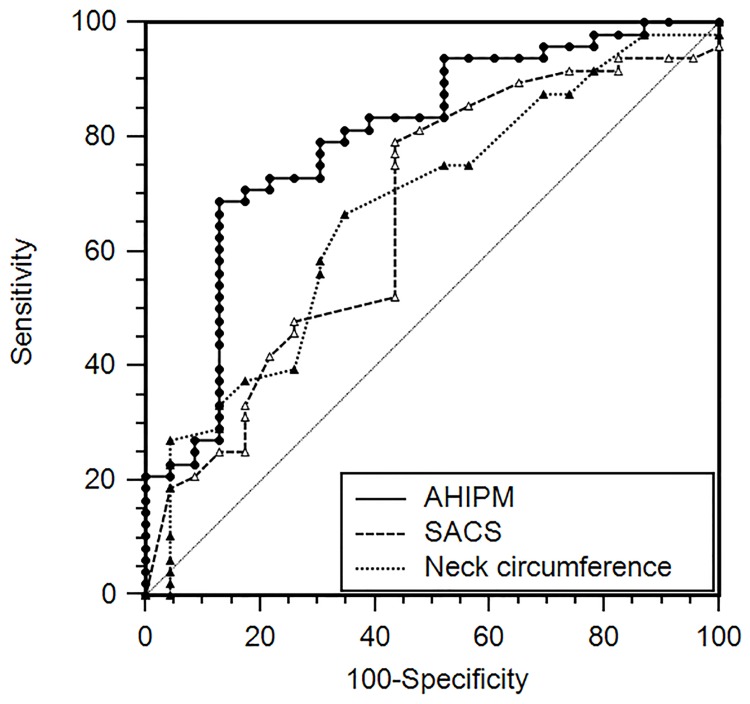
Receiver operating characteristic curve for AHI_PM_, Sleep Apnea Clinical Score, and neck circumference for predicting AHI_PSG_ ≥ 15. The areas were similar for the three predictors.

**Fig 4:**
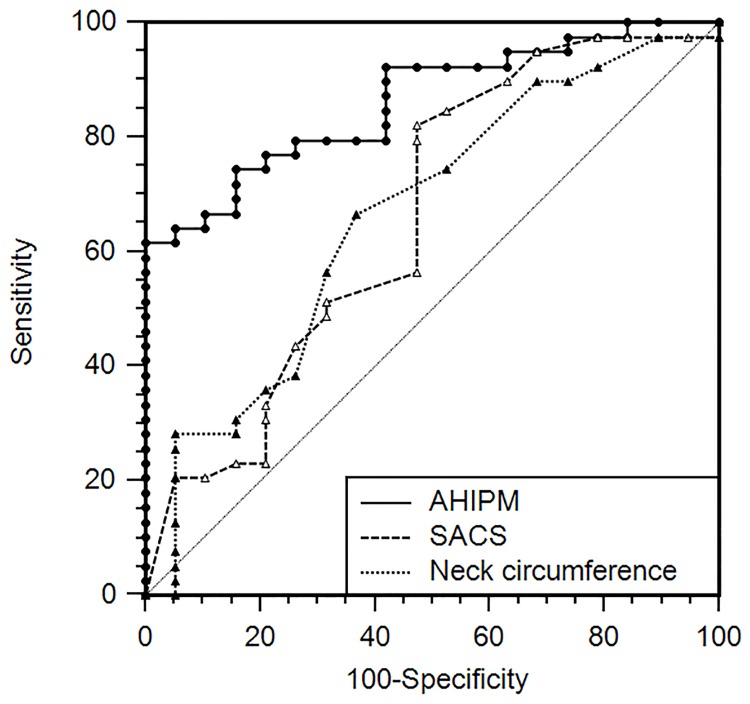
Receiver operating characteristic curve for AHI_PM_, Sleep Apnea Clinical Score, and neck circumference for predicting AHI_PSG_ ≥ 15, excluding patients with central apnea index ≥ 5 on portable monitoring. In these cases, AHI_PM_ was more accurate than the Sleep Apnea Clinical Score and neck circumference.

The AU-ROC was similar between AHI_PM_, neck circumference (0.67±0.07), and SACS (0.66±0.07) for detecting subjects with AHI_PSG_ ≥ 15 (Fig 3). After excluding the 13 subjects with CAI_PM_ ≥ 5, the AU-ROC curve of AHI_PM_ for detecting subjects with AHI_PSG_ ≥ 15 increased to 0.86 and was significantly larger than the AU-ROC for both neck circumference and SACS (Fig 4).

The effect of central apneas on the ability of PM in predicting AHI_PSG_ was further evaluated with linear regression. The linear regression equation to predict AHI_PSG_ from the portable indices was AHI_PSG_ = [0.9×AHI_PM_]-[1.7×CAI_PM_] with an R-squared of 0.70.

#### The influence of BMI and the Epworth sleepiness score

The area under the ROC curve did not change significantly in subgroups based on obesity and sleepiness. In patients with BMI ≥ 40, the optimal threshold for predicting AHI_PSG_≥ 30 was an AHI_PM_ of 18 (AUC = 0.80); for patients with a BMI < 40, the optimal threshold was 22 (AUC = 0.85). In patients with an Epworth sleepiness score ≥ 15, the optimal AHI threshold for predicting AHI_PSG_≥ 30 was 32 (AUC = 0.85); for patients with an Epworth ≤ 14, the optimal threshold was 21 (AUC = 0.82).

### Correlation and Agreement

**Fig 5:**
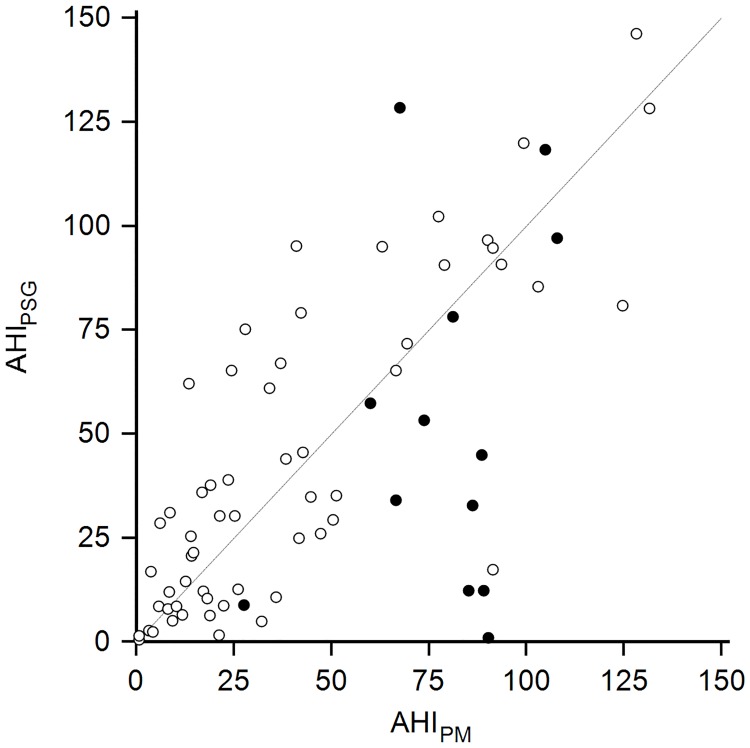
Scatterplot for AHI_PM_ and AHI_PSG_. The line represents perfect agreement. The black circles represent patients with central apnea index on portable monitoring ≥ 5.

**Fig 6:**
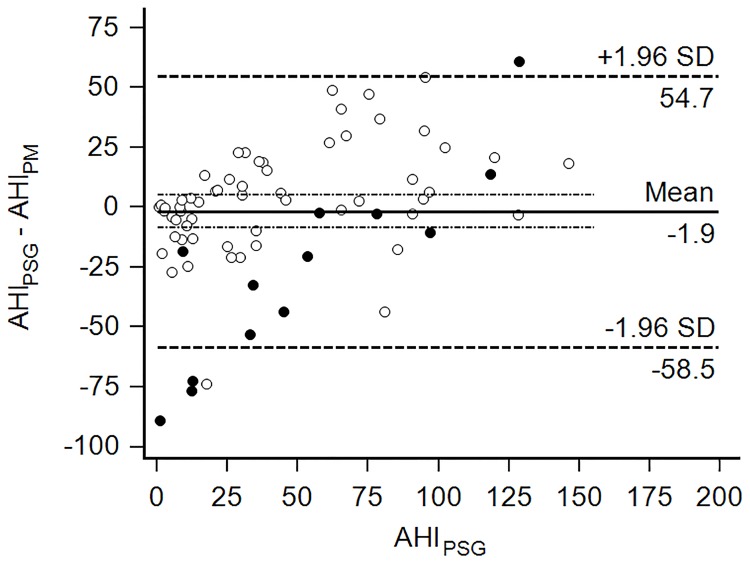
Modified Bland-Altman plot for AHI_PM_ and AHI_PSG_. The difference between AHI_PSG_ and AHI_PM_ was plotted against AHI_PSG_. Dark circles represent cases in which the central apnea index on portable monitoring was ≥ 5.

The intraclass correlation and the Spearman’s correlations coefficients between the AHI_PM_ and the AHI_PSG_ (Fig 5) were 0.7 and 0.68, respectively (P < 0.0001 for both). The mean±SD difference between AHI_PM_ and AHI_PSG_ was 2±29 in all patients and 4±22 in patients with CAI_PM_<5. The Modified Bland-Altman plot illustrates the distribution of the difference between AHI_PM_ and the AHI_PSG_ (Fig 6). Visual inspection indicates that the AHI_PM_ is lower than AHI_PSG_ when the latter is more than 50. When the AHI_PSG_ is less than 50, AHI_PM_ is higher than AHI_PSG_ in patients with CAI_PM_≥5.

## Discussion

In this study we found that PM is accurate in detecting moderate and severe SDB in hospitalized patients. The accuracy of PM was better in patients that did not have significant CSA during the hospitalization.

PM has not been the preferred test for diagnosing SDB in patients with comorbidities.[23] Its diagnostic accuracy when it is performed at home had been usually evaluated in patients without comorbidities.[28,29] However, Guerrero and colleagues reported that a 3-night PM was also accurate in ambulatory patients with mild comorbidities.[30] Our study expands on these results because the patients included in our study not only had more serious comorbidities, but they were also acutely ill; and yet, we were able to show that PM was as accurate in these patients as it is in stable patients tested at home.

The AHI_PM_ thresholds to predict each AHI_PSG_ threshold have different diagnostic priorities. When the objective was to identify AHI_PSG_ >15, the priority was specificity. That is why the optimal AHI_PM_ threshold was 36. On the other hand, when the objective was identifying patients with AHI_PSG_> 30, the priority was sensitivity. That is why the AHI_PM_ threshold was 22. In both cases, the selected threshold maximized the Youden index.

The sensitivity, specificity, likelihood ratios, and positive and negative predictive values in our study are similar to those reported by Masa and colleagues for home sleep testing with similar portable monitors.[28] Others have reported much higher accuracy of PM in the diagnosis of moderate to severe SDB in ambulatory patient. This high level of accuracy in these studies is from performing the PM and PSG simultaneously as in the study by Gantner and colleagues.[31] By doing so, the day to day variability is eliminated.

The AHI_PM_ thresholds identified in this study can guide in the choice between prescribing CPAP before a patient is discharged or recommending an outpatient PSG. For example, it is probably appropriate to discharge non-hypercapnic patients with AHI_PM_ ≥32 on CPAP. And as is the case of home testing, patients with an AHI_PM_<15 probably require an outpatient PSG.[29]

Obesity hypoventilation is common in obese hospitalized patients. The mortality of these patients in the first three months after discharge is high if they are discharged without treatment.[32] Therefore, waiting for outpatient testing might delay lifesaving therapy. Forty-four percent of our patients had hypercapnia. It is therefore reasonable to measure arterial blood gases in all patients suspected of having SDB during a hospitalization because the presence of hypercapnia increase the importance of discharging the patient on therapy. In the hypercapnic patients, an AHI_PM_ ≥ 19 is sufficiently accurate to diagnose SDB and prescribe therapy that will improve long-term outcomes.[33] Although these patients might require non-invasive ventilation during the hospitalization to correct acute on chronic respiratory failure, CPAP and noninvasive ventilation has similar effects on PCO_2_ in the ambulatory setting.[34]

During the hospitalization, we observed central sleep apnea in 18% of our patients. The CAI decreased significantly after the hospitalization especially in patients with low ejection fraction. This probably explains why PM was more accurate in patients with CAI_PM_<5. Although central sleep apnea is less amenable to treatment with CPAP, clinicians must be aware of its prognostic and therapeutic implications in patients with heart failure.[35,36]

The patients included in this study are likely to benefit from treatment of SDB. We focused in this study on the ability of PM to diagnose moderate and severe SDB. Evidence from cohort studies suggests that moderate SDB, not mild SDB, is associated with cardiovascular disease and that only severe SDB increases long-term mortality.[37] It is also important to note that most of the patients included in this study were sleepy. Prescribing CPAP to non-sleepy patients with coronary artery disease and SDB did not significantly reduce cardiovascular complications in a recently published study.[38]

We found that neck circumference and SACS were predictive of significant SDB. Other clinical tools used for screening ambulatory patients were not as accurate in high risk or hospitalized patients. For example, the sensitivity and specificity of the Berlin Questionnaire were low in patients with a recent myocardial infarction.[39] Similarly, the Cleveland Sleep Habits Questionnaire did not distinguish patients with SDB from patients without SDB during a hospitalization for acute coronary syndrome.[17] Although they do not confirm a diagnosis of SDB, the neck circumference and SACS might help prioritize testing during a hospitalization.

The limits of agreement on AHI were 57 above and below the difference between AHI_PSG_ and AHI_PM_ but decreased to 43 in patients with CAI_PM_<5. This is similar to the limits of agreement for home testing with portable devices and probably reflects differences between testing methods, patient condition, as well as night to night change in AHI.[28,40,41]

Because we educated the hospital staff to help ensure adequate testing, the PM during a hospitalization can be considered as a partially attended test.[28,29] This possibly explains the low failure rate of PM during a hospitalization in comparison to home testing. In the study by Kauta and colleagues, only 2 of 106 hospitalized patients had inconclusive studies because of multiple lead failures.[14] In contrast, Masa and colleagues had to repeat 24% of the home studies to obtain valid data.[42] And although we defined adequate PM as a study with 3 hours of acceptable scored recording that included oximetry and either a flow or an effort signal, the average scored recording time was over 7 hours.

Our study has multiple strengths. First, we attempted to maximize the accuracy of the AHI_PM_ by using actigraphy to measure sleep, measuring flow by 2 methods, and having a physician score the studies.[23,43] Actigraphy is not yet a standard element in PM studies. It was useful for the investigators in selecting the main sleep period and in estimating sleep efficiency. Yet, we cannot extrapolate our results to portable monitoring without actigraphy, measuring flow with 1 method, or relying on automated scoring. Second, the physicians reviewing the PSGs and the PMs were blinded to the results of the other tests. Third, this study is representative of standard practice because the hospitalized patients were not screened by us but were referred to the study when their hospitalists suspected SDB. Fourth, we defined hypopneas using the 4% desaturation (acceptable AASM definition) and not the standard 3% definition because it is the one associated with cardiovascular outcomes.[44] This was important for our study, because ultimately, reducing cardiovascular outcomes and mortality is the goal of identifying SDB in hospitalized patients.

Our study has its limitations. First, this was a single center cohort with a specific ethnic (predominantly African-American) and economic status (uninsured and underinsured) and a high pretest probability for SDB. Even so, the effect of severe untreated severe SDB on health may be independent from ethnicity and insurance. Second, the no-show rate was higher than expected (52%). This reduced the power of the study to 0.73 from the desired 0.9. These patients had similar age, sex, and BMI to the patients that had the PSG. Similar rates of missed PSG appointments have been reported by other investigators.[45,46] The high rates of missed appointments underscore the value of diagnosing and treating SDB during hospitalizations and not deferring the evaluation to the outpatient setting. [20] Third, there was a 3 month delay between the PM during the hospitalization and the outpatient PSG. Although there is day to day variation in respiratory indexes of SDB, this variation may not affect the positive rate of the outpatient PSG because of our focus on identifying severe SDB.

In conclusion, PM is accurate in detecting moderate and severe SDB in high risk hospitalized patients. It also identifies patients with central sleep apnea who might need outpatient PSG once they are optimally treated for heart failure and other comorbidities to determine the nature of their underlying SDB. Therefore, PM should be used more frequently during hospitalizations to detect significant SDB and prescribe long-term CPAP because such a strategy could reduce readmission and mortality. [10,14]

## Supporting Information

S1 Dataset Copy of Data for Submission. (XLS)
